# Interleukin 6 exacerbates the progression of warm autoimmune hemolytic anemia by influencing the activity and function of B cells

**DOI:** 10.1038/s41598-023-40239-w

**Published:** 2023-08-14

**Authors:** Manjun Zhao, Lei Chen, Jin Yang, Ziying Zhang, Huaquan Wang, Zonghong Shao, Xiaoqing Liu, Limin Xing

**Affiliations:** 1 Division of Infectious Diseases, Department of Internal Medicine, Peking Union Medical College Hospital, Chinese Academy of Medical Sciences and Peking Union Medical College, Beijing, 100730 China; 2 Department of Hematology, Tianjin Medical University General Hospital, Tianjin, 300052 China

**Keywords:** Autoimmunity, Cytokines, B cells

## Abstract

To explore the effect of IL-6 on the activity and secretory function of B cells and analyze its effect on clinical indicators and efficacy in wAIHA patients. This study included 25 hemolytic wAIHA patients, 13 remission patients, and 10 HCs. Plasma levels of various cytokines were detected using CBA. PBMCs were extracted from 12 hemolytic wAIHA patients and divided into three wells, stimulation with IL-6 and IL-6 + tocilizumab, the blank control wells were also set. After 48 h of in vitro cell culture, percentage of CD5^+^CD80^+^, CD5^–^CD80^+^,CD5^+^CD86^+^,CD5^–^CD86^+^,CD5^+^IL-10^+^,CD5^–^IL-10^+^B cells were determined by flow-cytometry. Plasma levels of IL-6 and IL-10 in hemolytic episode group were significantly higher than that in HCs group (*p* = 0.0243; *p* = 0.0214). RBC and Hb levels were negatively correlated with IL-6 levels in wAIHA patients, while LDH levels were positively correlated.Therapeutic effects of glucocorticoid and duration of efficacy were also significantly correlated with IL-6 levels in wAIHA patients. After 48 h in vitro cell culture, percentages of CD80^+^/CD5^+^CD19^+^and CD80^+^/CD5^–^CD19^+^ cells in the IL-6 stimulation group were higher than those in blank control group (*p* = 0.0019; *p* = 0.0004), while CD86^+^/CD5^+^ CD19^+^ and CD86^+^/CD5^–^CD19^+^ cells were not statistically different before and after IL-6 stimulation. Percentage of IL-10^+^/CD5^+^ CD19^+^ cells in IL-6 stimulation group was lower than that in blank control (*p* = 0.0017) and IL-6 + toc (*p* = 0.0117) group. Percentage of IL-10^+^/CD5^–^ CD19^+^cells in the IL-6 stimulation group was lower than that in the blank control group (*p* = 0.0223). Plasma levels of IL-6 were significantly elevated in hemolytic wAIHA patients and correlated with clinical indicators and efficacy. IL-6 promotes the activation of B cells. Although the results were not statistically significant, IL-6R antagonist tocilizumab may hopefully become a targeted therapy for wAIHA patients.

## Introduction

Autoimmune Hemolytic Anemia (AIHA) is a relatively rare and heterogeneous autoimmune disease (AID) characterized by auto-antibodies against red blood cell (RBC) surface antigens generated and secreted by hyperactive B lymphocytes. According to the binding properties of auto-antibodies, AIHA can be further divided into warm AIHA (wAIHA), cold AIHA, and mixed-type AIHA^1,2^. The most common type is wAIHA, which accounts for 70–75% of all AIHA cases. Several factors contribute to this rare AID, including antibody-dependent cellular cytotoxicity and the dysregulation of cytokines, such as interleukin (IL)-4, IL-6, and IL-10^3^.

IL-6 was initially identified as a cytokine that promotes the activation and differentiation of B cells and has subsequently been found to regulate various biological processes, including immune response, hematopoiesis, and acute inflammation reactions^4^. IL-6 can also enhance the function of CD4^+^ T cells to assist B cell activation by promoting the production of IL-21, therefore stimulating the humoral immune response. Elevated levels of IL-6 have been reported to correlate with auto-antibodies production^5^. Overactivation of the IL-6 signaling pathway can also induce the expression of the pre-B-cell colony-enhancing factor (PBEF), which plays an important role in the progression of rheumatoid arthritis (RA)^6^. IL-6 seems to prompt the progression of AID by mediating B-cell hyper immunity and the secretion of auto-antibodies.

Our previous study revealed that the expression levels of the activation molecules CD80 and CD86 on the surface of CD5^+^B cells were significantly elevated and correlated with the hemolytic and immune indicators in AIHA patients^7^. Activated CD5^+^B cells in AIHA patients mainly secrete IL-10 which negatively regulate the immune response^8^. Therefore, we hypothesized that IL-6 plays a role in AIHA patients by regulating the activity and secretory function of B cells, especially CD5^+^ B cells. In this study, we determined, using Cytometric bead array (CBA), the expression levels of various cytokines reported to participate in the immune-inflammation process, including IL-6 in wAIHA patients, and analyzed the relationship between IL-6 levels and clinical indicators or efficacy. Peripheral blood mononuclear cells (PBMCs) from hemolytic wAIHA patients were isolated and cultured in vitro and stimulated with IL-6 and IL-6 + tocilizumab, respectively. Tocilizumab is a humanized monoclonal antibody that acts as an IL-6 receptor (IL-6R) antagonist. After 48 h of in vitro cell culture, the percentage of CD5^+^CD80^+^,CD5^+^CD86^+^,CD5^+^IL-10^+^B cells for each group were detected by flow cytometry to explore the effects of IL-6 on peripheral blood B cells’ activity and secretory function in wAIHA patients.

## Methods and materials

### Study subjects

The study subjects were patients with wAIHA hospitalized at Hematology Department of Tianjin Medical University General Hospital, China, from November 2019 to March 2022. All wAIHA patients were diagnosed based on the Chinese expert consensus on the diagnosis and treatment of autoimmune hemolytic anemia (2017 edition)^9^.

The diagnostic criteria were as follows: (1) Hemoglobin (Hb) level meeting the diagnostic criteria for anemia (males < 120 g/L; females < 110 g/L); (2) Auto-antibodies against RBC were detected in patients; (3) The results of laboratory tests met at least one of: (i) The percentage of reticulocytes (Ret%) was > 4% or absolute value > 120 × 10^9^/L, or (ii) Haptoglobin (Hp) < 100 mg/L. Total bilirubin (TBIL) ≥ 17.1 µmol/L and indirect bilirubin (IBIL) are mainly elevated. AIHA was also diagnosed if patients responded well to glucocorticoid but had a negative Coombs test.

Patients enrolled in this study included 25 hemolytic episode patients with wAIHA (10 males and 15 females, with a median age of 49 [19–77] years), 13 patients in remission (6 males and 7 females, with a median age of 63 [17–74] years), and 10 healthy controls (HCs) (4 females and 6 males, with a median age of 49 [15–68] years). All study participants provided written informed consent, and the study design was approved by the Ethics Committee of Tianjin Medical University General Hospital and was performed in accordance with the Declaration of Helsinki.

### Detection of plasma levels of cytokines in wAIHA patients and HCs by CBA

Exactly 10 ml of EDTA anticoagulant peripheral venous blood was obtained from each subject and centrifuged at *400 g* for 10 min. The plasma was extracted, and the levels of IL-2, IL-4, IL-6, IL-10, TNF-α, and IFN-γ were determined according to the Luminex human cytokines plex detection instruction manual kit (BD Bioscience Company, US).

### Separation of PBMCs and in vitro cell culture

**Figure 1:**
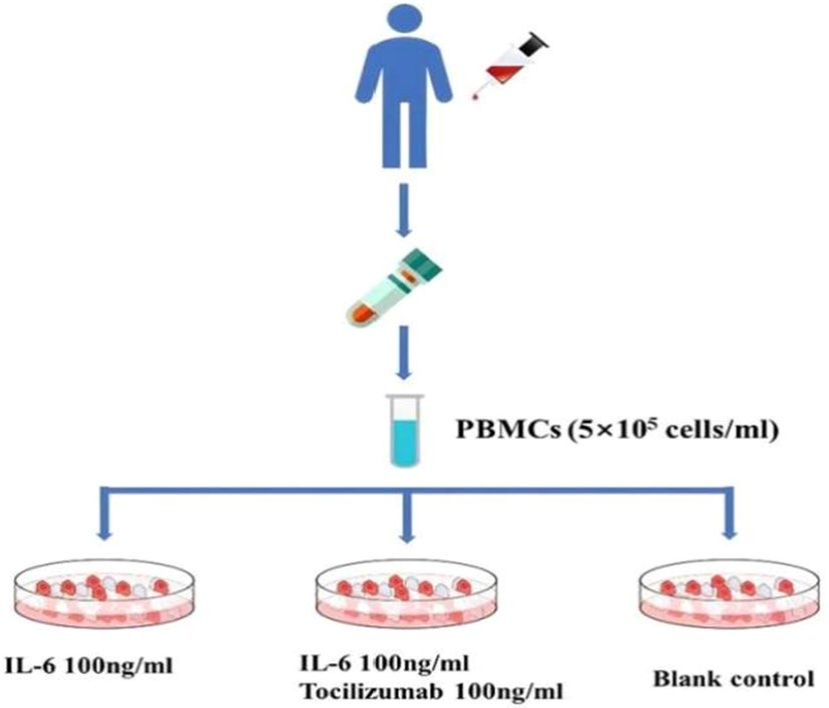
Peripheral blood mononuclear cells (PBMCs) were extracted from 12 hemolytic wAIHA patients. Cells were transferred to 24-well cell culture plates. 100 ng/mL of IL-6 or 100 ng/mL of IL-6 + tocilizumab was added, respectively. Blank control well was also set. PBMCs cultured in vitro for 48 h at 37 °C and 5% CO_2_.

Twelve blood samples of hemolytic wAIHA patients were randomly selected to perform in vitro cell culture.The rest of the blood specimen was fully mixed with equivalent volume of normal saline solution. The mixture was slowly layered on the top of the same amount of lymphocyte separation medium in a separate tube (Solarbio Science & Technology Co., Ltd. Beijing, China) and centrifuged at 600*g* for 20 min, and PBMCs were obtained. According to the cell counts, appropriate amount of cell culture medium (RPMI 1640 + 10% fetal bovine serum + 1% Penicillin–Streptomycin Liquid) were added to adjust the concentration to 5 × 10^5^ /ml. Cells were transferred to 24-well cell culture plates. Blank control well, IL-6 stimulation well, and IL-6 + tocilizumab (toc) stimulation well was set separately for each sample. Exactly 1 ml of cell suspension was added to each well, and 100 ng/ml of IL-6 (200-06, PeproTech Bioscience Company, US) or 100 ng/ml IL-6 + 100 ng/mL toc (HY-P9917, MedChem Express Bioscience Company, US) was added. PBMCs were cultured in vitro for 48 h at 37 °C and 5% CO_2_ (Fig. 1).

### Detection of the percentage of CD80^+^/CD5^+^CD19^+^, CD86^+^/CD5^+^CD19^+^, CD80^+^/CD5^–^ CD19^+^, CD 86^+^/CD5^–^CD19^+^ B cells by flow cytometry

After incubation for 48 h, PBMCs were collected and washed with 10 ml sterile phosphate- buffered saline (PBS) at 400*g* for 5 min. After discarding the supernatant, cells were re-suspended in 1 ml PBS again. Three test tubes were set for each sample. 5 µl PCY7-CD19 (557,835, BD), 5 µl APC-CD5 (555,355, BD) and 100 µl cell suspension were added in each tube. Then the samples were complemented with 5 µl PE-IgG1 (349,043, BD), 5 µl PE-CD80 (12-0809-42, eBioscience) or 5 µl PE-CD86 (12-0869-42, eBioscience), respectively. The amount of antibody used in flow-cytometry is based on the recommendation by the company. The mixture was mixed thoroughly and incubated for 15 min in the dark. PBS (1 ml) was then added to each test tube and mixed thoroughly. The mixture was centrifuged at 400*g* for 5 min, and the supernatant was discarded. Exactly 300 µL of PBS was added to re-suspend the cells in each test tube again, and the cells were detected by flow cytometry (Beckman Coulter Co., Ltd., Germany) and analyzed using CytExpert software.

### Detection of percentage of IL-10^+^/CD5^+^CD19^+^ and IL-10^+^/CD5^–^CD19^+^ B cells by flow cytometry

The cell suspensions (300 µl) were obtained after 48 h in vitro cell culture, and 1 µl of Leukocyte Activation Cocktail (BD Bioscience Company, US) was added to stimulate the cells. The cells were incubated for 4 − 6 h at 37 °C and 5% CO_2_. Two test tubes were set for each sample. Exactly 5 µL of PCY7-CD19 (557,835, BD), 5µL of APC-CD5(555,355, BD), and 100 µL of the cell suspensions were added to each tube. After mixing thoroughly, the tubes were incubated at room temperature in the dark for 15 min. Exactly 100 µL IntraSure™ Reagent A were added into each tube, mixed well, and left to stand for 5 min at room temperature. PBS buffer (1 mL) were added, and the mixtures were centrifuged at 400*g* for 5 min. The supernatant was then discarded. Exactly 40 µL of IntraSure™ Reagent B and 5 µL of PE-IgG1(349,043, BD) or PE-IL-10 (12-7108-82, eBioscience) were added to each test tube. After mixing, the mixtures were incubated for 15 min at room temperature in the dark. Exactly 1 ml of PBS were added to the two test tubes, mixed thoroughly, and centrifuged for 5 min at 400*g*. The supernatant was discarded, and 300 µL of PBS were added to re-suspend the cells again. The cells were detected by flow cytometry (Beckman Coulter Co., LTD., Germany) and analyzed using CytExpert software.

B cells can not be divided clearly into two cell subsets when labeled with the fluorescent antibody APC-CD5, PE-CD80, PE-CD86 and PE-IL-10. We used isotype control antibodies IgG1-PE to help us distinguishing positive cells from negative cells. (See Supplementary Materials).

### Statistical analysis

All data analyses were performed using SPSS 22.0. The results were expressed as the mean ± standard deviation. Comparisons between two groups were analyzed using Student’s *t* test. One-way analysis of variance was used for several independent groups. Pearson correlation analysis was used for correlation analysis between numerical variables, and Kendall correlation analysis was used for correlation analysis between numerical and classification variables.Statistical differences were considered statistically significant at *p* < 0.05. GraphPad Prism 8 software was used to draw all statistical analyses.

## Results

### Plasma levels of cytokines in patients with wAIHA and HCs

**Figure 2:**
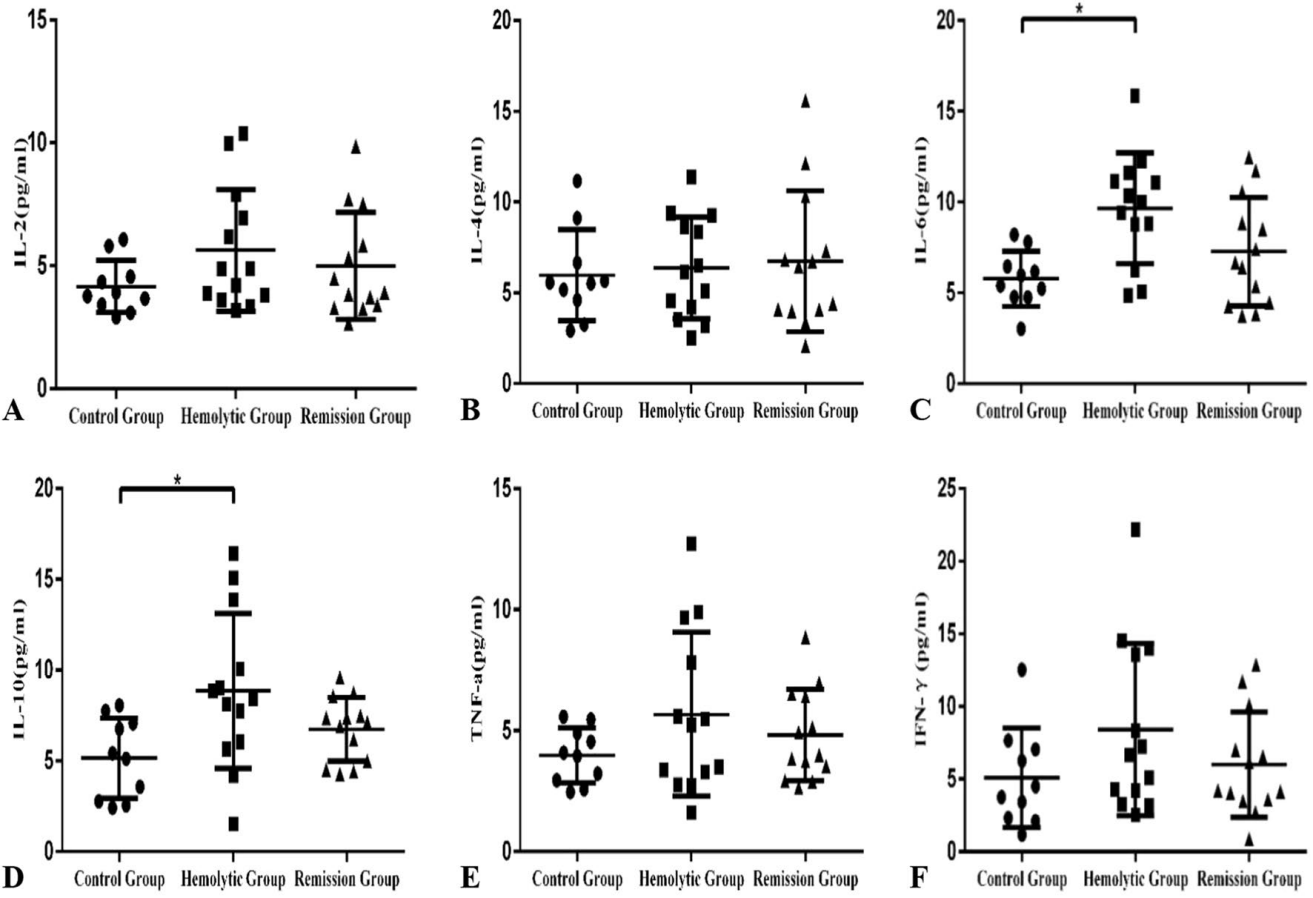
Plasma levels of IL-6 (**C**) and IL-10 (**D**) in hemolytic episode group (9.04 ± 1.11 pg/mL, 8.85 ± 1.18 pg/mL) were significantly increased compared to HC group (5.78 ± 0.48 pg/mL, *p* = 0.0243; 5.15 ± 0.70 pg/mL, *p* = 0.0214) respectively.Plasma levels of IL-2 (**A**), IL-4 (**B**), TNF-α (**E**), and IFN-γ (**F**) were not significantly different among three groups.

The plasma levels of IL-6 in hemolytic wAIHA patients (9.66 ± 0.84 pg/mL) were significantly higher than that of HCs (5.78 ± 0.48 pg/mL, *p* = 0.0243). The differences between hemolytic wAIHA patients and remission patients (7.27 ± 0.83, *p* = 0.0549) or between remission patients and HCs (*p* = 0.1677) were not statistically significant (Fig. 2C). The plasma levels of IL-10 in hemolytic wAIHA patients (8.85 ± 1.18 pg/mL) were significantly higher than that of HCs (5.15 ± 0.70 pg/mL, *p* = 0.0214). The differences between hemolytic wAIHA patients and remission patients (6.75 ± 0.49, *p* = 0.1128) or between remission patients and HCs (*p* = 0.0669) were not statistically significant(Fig. 2D). Plasma levels of IL-2(Fig. 2A), IL-4(Fig. 2B), TNF-α(Fig. 2E), and IFN-γ(Fig. 2F) were not significantly different among three groups (Table 1).

**Table 1:** Plasma levels of cytokines in wAIHA patients and HCs.

	Hemolytic episode group	Remission group	Healthy controls
IL-2 (pg/mL)	5.63 ± 0.69	4.99 ± 0.60	4.16 ± 0.34
IL-4 (pg/mL)	6.37 ± 0.78	6.74 ± 1.08	5.97 ± 0.80
IL-6 (pg/mL)	9.66 ± 0.84*	7.27 ± 0.83	5.78 ± 0.48
IL-10 (pg/mL)	8.85 ± 1.18*	6.75 ± 0.49	5.15 ± 0.70
TNF-α (pg/mL)	5.67 ± 0.94	4.81 ± 0.53	3.98 ± 0.36
IFN-γ (pg/mL)	8.41 ± 1.65	5.98 ± 1.00	5.08 ± 1.08

Compared with healthy controls.

*AIHA* autoimmune hemolytic anemia, *HC* healthy controls.

*p < 0.05.

### Correlation analysis between plasma levels of IL-6 with clinical indicators or efficacy of wAIHA patients

**Figure 3:**
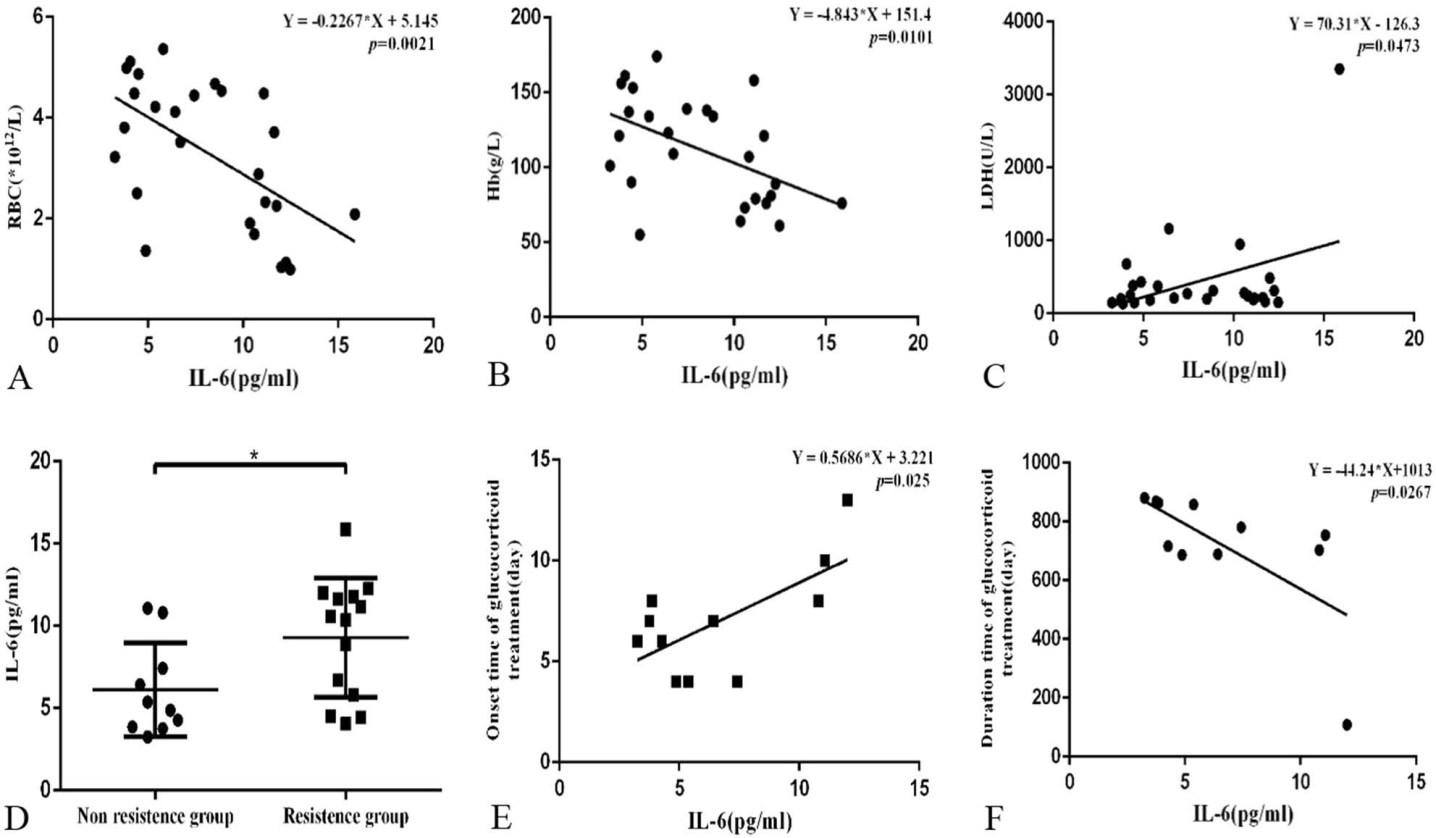
RBC count (**A**) (r = – 0.5745, *p* = 0.0021) and Hb level (**B**) (r =  − 0.4954, *p* = 0.0101) were negatively correlated with IL-6 in wAIHA patients.The level of LDH (**C**) (r = 0.3926, *p* = 0.0473) was positively correlated with IL-6 level in wAIHA patients. Plasma levels of IL-6 were significantly higher in glucocorticoid-resistant wAIHA patients (9.28 ± 0.98 pg/mL) compared to patients who responded effectively to glucocorticoid therapy (6.11 ± 0.90 pg/mL, *p* = 0.0312) (**D**).Onset time for glucocorticoid treatment was positively correlated (**E**) (Y = 0.5686X + 3.221, r = 0.667, *p* = 0.025), and duration of glucocorticoid treatment was negatively correlated with plasma level of IL-6 (**F**) (Y = − 44.24X + 1013, r =  − 0.661, *p* = 0.0267) in hemolytic wAIHA patients.

RBC counts (r = − 0.5745, *p* = 0.0021) (Fig. 3A)and Hb levels (r = − 0.4954, *p* = 0.0101) (Fig. 3B) were negatively correlated, and LDH levels (r = 0.3926, *p* = 0.0473) (Fig. 3C) were positively correlated with IL-6 levels in wAIHA patients.We followed up the therapeutic effects of glucocorticoids in hemolytic AIHA patients.Patients who received dosage of 0.5–1.5 mg/Kg/day prednisone for more than 4 weeks didn’t achieved clinical remission were considered to be glucocorticoid-resistant patients. Therefore, hemolytic AIHA patients can be furthered divided into two groups, glucocorticoid-resistant group and non-resistant group. The plasma levels of IL-6 were significantly higher in glucocorticoid-resistant wAIHA patients (9.28 ± 0.98 pg/mL) compared to patients who responded effectively to glucocorticoid therapy (6.11 ± 0.90 pg/mL, *p* = 0.0312) (Fig. 3D).

**Figure 4:**
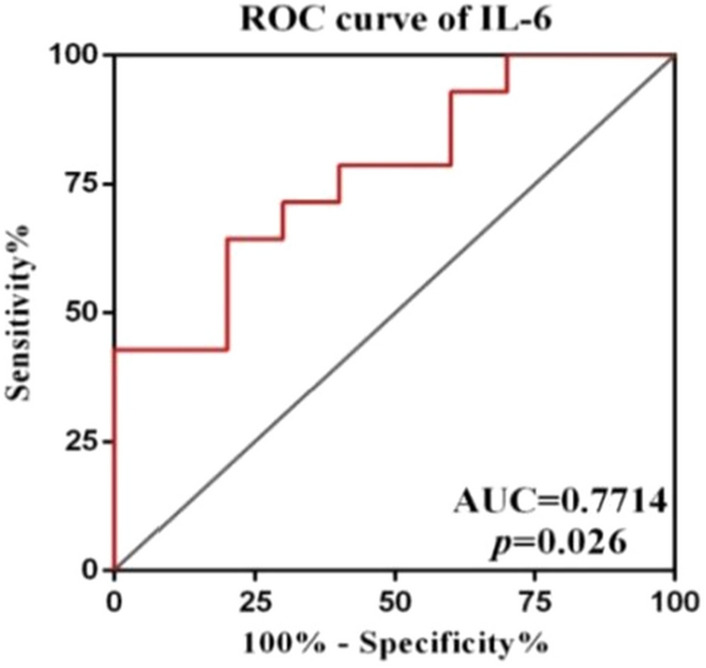
ROC curve analysis for IL-6 to predict the effects of glucocorticoid therapy. The best cut-off value was 8.145 pg/ml with the sensitivity and specificity of 64% and 80% respectively.

The relationship between the plasma levels of IL-6 and the therapeutic efficacy of glucocorticoid in wAIHA patients were analyzed.The receiver operating characteristic (ROC) curve generated by SPSS software has been shown in Fig. 4. The best cut-off value was 8.145 pg/ml with the the sensitivity and specificity of 64% and 80% respectively.

The onset time for glucocorticoid treatment was positively correlated (Y = 0.5686X + 3.221, r = 0.667, *p* = 0.025) (Fig. 3E), and the duration of glucocorticoid treatment was negatively correlated with plasma levels of IL-6 (Y = − 44.24X + 1013, r = -0.661, *p* = 0.0267) (Fig. 3F)in hemolytic wAIHA patients. There was no significant relationship between disease relapse and plasma levels of IL-6 in wAIHA patients (*p* > 0.05).

### The percentage of CD80^+^/CD5^+^CD19^+^, CD86^+^/CD5^+^CD19^+^, CD80^+^/CD5^–^CD19^+^, CD 86^+^/CD5^–^CD19^+^B cells after in vitro cell culture

**Figure 5:**
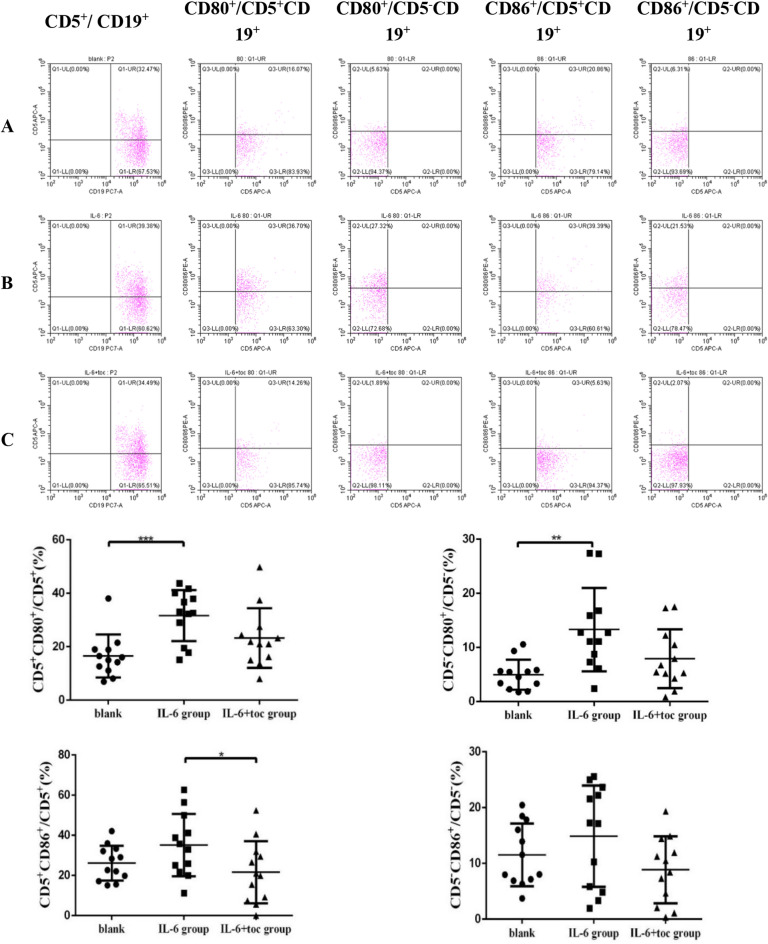
After 48 h in vitro cell culture, the percentage of CD5^+^ B cells shows no significant differences among blank control (**A**), IL-6 stimulation group (**B**) and IL-6 + toc group (**C**). The percentage of CD5^+^CD80^+^B cells and CD5^-^CD80^+^B cells were significantly elevated in IL-6 stimulation group.

After 48 h in vitro cell culture, no statistically significant difference was observed in the proportion of CD19^+^B cells among IL-6 stimulation (Fig. 5B), IL-6 + toc stimulation (Fig. 5C), and blank control groups (Fig. 5A) [(18.69 ± 1.80, 17.08 ± 2.41 and 14.72 ± 1.16)%]. There was no statistically significant difference in the proportion of CD5^+^/CD19^+^B cells between the IL-6 stimulation group [(28.73 ± 4.65)%] (Fig. 5B), the IL-6 + toc stimulation group [(25.17 ± 4.07)%] (Fig. 5C) and the blank control group [(25.30 ± 4.50)%] (Fig. 5A). Percentage of CD80^+^/CD5^+^CD19^+^ B cells in each group were detected. The proportion of CD80^+^/CD5^+^CD19^+^ B cells in the IL-6 stimulation group [(31.63 ± 2.76)%] (Fig. 5B) was significantly higher than that in the blank control group [(16.53 ± 2.33)%, *p* = 0.0004] (Fig. 5A), but was not significantly different from that in IL-6 + toc stimulation group [(23.26 ± 3.23)%] (Fig. 5C). Proportion of CD80^+^/CD5^–^CD19^+^ B cells in each group were detected. Proportion of CD80^+^/CD5^–^CD19^+^ B cells in the IL-6 stimulation group [(13.31 ± 2.22)%] (Fig. 5B) was significantly higher than that in the blank control group [(4.98 ± 0.80)%, *p* = 0.0019] (Fig. 5A), but was not significantly different from that in the IL-6 + toc stimulation group [(7.93 ± 1.57)%] (Fig. 5C). Although the change was not statistically significant, the percentage of CD80^+^/CD5^+^CD19^+^ and CD80^+^/CD5^-^CD19^+^ B cells in the IL-6 + toc stimulation group showed a decreasing trend compared to the IL-6 stimulation group (Table 2).

**Table 2:** Percentage of CD5^+^CD19^+^,CD80^+^/CD5^+^CD19^+^,CD80^+^/CD5^–^CD19^+^,CD86^+^/CD5^+^CD19^+^,CD86^+^/CD5^–^CD19^+^ B cells after 48 h in vitro cell culture detected by flow cytometry.

(%)	Blank control group	IL-6 stimulation group	IL-6 + toc stimulation group
CD19^+^	14.72 ± 1.16	18.69 ± 1.80	17.08 ± 2.41
CD5^+^/CD19^+^	25.30 ± 4.50	28.73 ± 4.65	25.17 ± 4.07
CD80^+^/CD5^+^CD19^+^	16.53 ± 2.33	31.63 ± 2.76***	23.26 ± 3.23
CD80^+^/CD5^–^CD19^+^	4.98 ± 0.80	13.31 ± 2.22**	7.93 ± 1.57
CD86^+^/CD5^+^CD19^+^	26.14 ± 2.52	35.12 ± 4.48	21.61 ± 4.45
CD86^+^/CD5^–^CD19^+^	11.54 ± 1.62	14.88 ± 2.62	8.86 ± 1.73

Compared with blank control group.

**p < 0.01, ***p < 0.001.

The proportion of CD86^+^/CD5^+^CD19^+^ B cells in each group were detected. The proportion of CD86^+^/CD5^+^CD19^+^ B cells in IL-6 stimulation group [(35.12 ± 4.48) %] (Fig. 5B)was higher than that in the IL-6 + toc stimulation group [(21.61 ± 4.45)%, *p* = 0.0438] (Fig. 5C), but was not significantly different from that in the blank control group [(26.14 ± 2.52)%] (Fig. 5A). The proportion of CD86^+^/CD5^–^CD19^+^B cells in each group were detected. The proportion of CD86^+^/CD5^-^CD19^+^ B cells in the IL-6 stimulation group [(14.88 ± 2.62)%] (Fig. 5B) was not statistically different from that in the IL-6 + toc stimulation group [(8.86 ± 1.73)%] (Fig. 5C) or blank control group [(11.54 ± 1.62%)] (*p* > 0.05) (Fig. 5A). Although the effect was not statistically significant, the proportion of CD86^+^/CD5^+^CD19^+^ and CD86^+^/CD5^-^CD19^+^ B cells was higher in the IL-6 stimulation group than in the blank control group (Table 2).

### The percentage of IL-10^+^/CD5^+^CD19^+^and IL-10^+^/CD5^–^CD19^+^ B cells after in vitro cell culture

**Figure 6:**
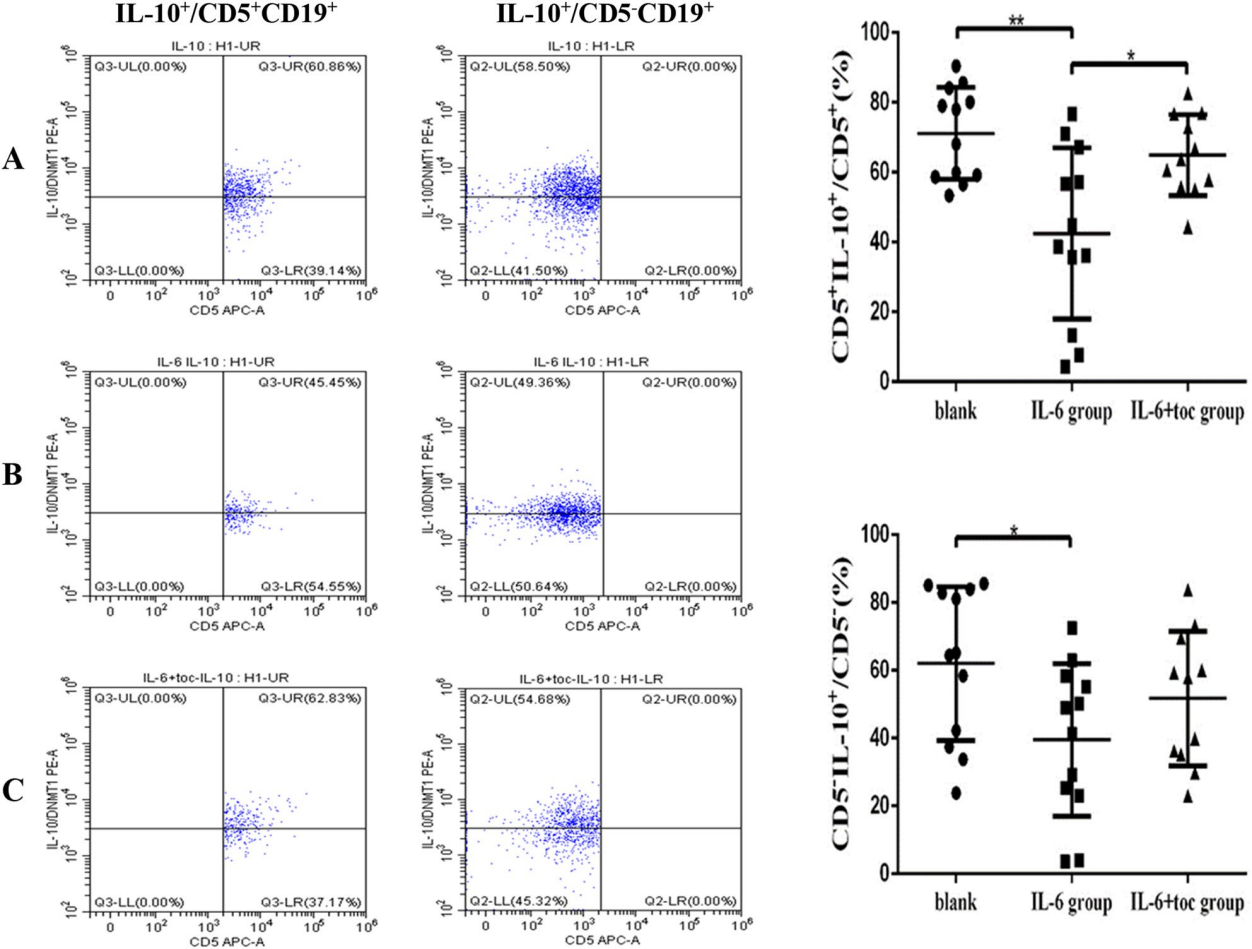
After 48 h in vitro cell culture, percentage of CD5^+^IL-10^+^ and CD5^–^IL-10^+^ B cells decreased significantly in IL-6 stimulation group (**B**) compared with blank control group (**A**). No statistically significant differences can be seen between blank control group (**A**) and IL-6 + toc group (**C**).

After 48 h of in vitro cell culture, the percentage of IL-10^+^/CD5^+^CD19^+^ B cells in each group were detected. The proportion of IL-10^+^/CD5^+^CD19^+^ B cells in the IL-6 stimulation group [(42.45 ± 7.08)%] (Fig. 6B) was significantly lower than in the control group [(71.12 ± 3.80)%, *p* = 0.0017] (Fig. 6A) and IL-6 + toc stimulation group [(64.89 ± 3.48)%, *p* = 0.0117] (Fig. 6C). The proportion of IL-10^+^/CD5^–^ CD19^+^ B cells in each group were detected. The proportion of IL-10^+^/CD5^–^CD19^+^ B cells in IL-6 stimulation group [(39.54 ± 6.49)%] (Fig. 6B) was significantly lower than that in blank control group [(62.01 ± 6.54)%, *p* = 0.0223] (Fig. 6A) but no statistically significant difference was observed with the IL-6 + toc stimulation group [(51.67 ± 5.98)%] (Fig. 6C). However, the proportion of IL-10^+^/CD5^–^ CD19^+^ B cells showed a decreasing trend in the IL-6 stimulation group compared to the IL-6 + toc stimulation group (Table 3).

**Table 3:** Percentage of IL-10^+^ B cells after 48 h in vitro cell culture detected by flow cytometry.

(%)	Blank control group	IL-6 stimulation group	IL-6 + toc stimulation group
IL-10^+^/CD5^+^CD19^+^	71.12 ± 3.80	42.45 ± 7.08**	64.89 ± 3.48
IL-10^+^/CD5^–^CD19^+^	62.01 ± 6.54	39.54 ± 6.49*	51.67 ± 5.98

Compared with blank control group.

*p < 0.05, **p < 0.01.

### Differences for the percentage of CD80^+^,CD86^+^ and IL-10^+^ cells between CD5^+^ and CD 5^–^ B cells after IL-6 stimulation

**Figure 7:**
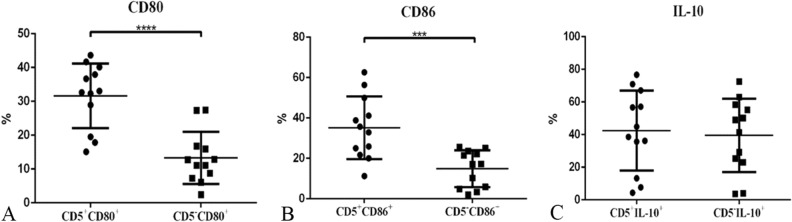
Percentage of CD5^+^CD80^+^B cells (**A**)and CD5^+^CD86^+^B cells (**B**) are significantly higher than CD5^–^ CD80^+^B cells and CD5^–^CD86^+^B cells respectively after IL-6 stimulation.Neither the percentage of CD5^+^IL-10^+^B cells nor the percentage of CD5^-^IL-10^+^ B cells was significantly altered by IL-6 stimulation (**C**).

The percentage of CD80^+^/CD5^+^CD19^+^ B cells [(31.63 ± 2.76)%] was significantly higher than the percentage of CD80^+^/CD5^–^CD19^+^ B cells in IL-6 stimulation group [(13.31 ± 2.22)%, *p* < 0.0001] (Fig. 7A). Proportion of CD86^+^/CD5^+^CD19^+^ B cells [(35.12 ± 4.48) %] was also significantly higher than proportion of CD86^+^/CD5^-^CD19^+^ [(14.88 ± 2.62)%, *p* = 0.0008] in IL-6 stimulation group (Fig. 7B). However, differences in the proportion of IL-10^+^ cells were not statistically significant between CD5^+^ and CD5^–^ B cells after IL-6 stimulation (*p* > 0.05) (Fig. 7C).

## Discussion

wAIHA is a rare AID mediated by auto-antibodies binding the surface antigens on RBC at body temperature. Studies have reported that B lymphocytes play an important role in the occurrence of many AID, not only by the production and secretion of auto-antibodies but also by cytokines secretion which promote the autoimmune response in our body. Similar to other AID, the development of wAIHA is associated with dysregulation of central and peripheral self-tolerance and the presence of auto-reactive T cells and B cells^10^.

Auto-antibodies against RBC surface antigens play an essential role in wAIHA pathogenesis. Our previous study demonstrated that B lymphocytes in patients with hemolytic wAIHA are abnormally activated with the over-expression of cell surface activation molecules CD80 and CD86^6^. However, the mechanism leading to B cell immune hyperfunction in patients is still unclear. Cytokines are soluble proteins produced by immune cells when stimulated by immunogens, mitogens or other factors. As molecules that transmit bio information, cytokines can regulate innate and adaptive immune responses, promote hematopoiesis, and induce immune cell activation, proliferation, and differentiation^11^. Cytokines play an important role in the pathogenesis of AID^12^.

In this study, various cytokines that have been reported to participate in the immune- inflammation process in wAIHA patients were detected by CBA. We found that IL-6 was significantly elevated in hemolytic episode wAIHA patients compared with remission patients and HCs. RBC counts and Hb levels were significantly negatively correlated, and the LDH level was significantly positively correlated with plasma level of IL-6 in wAIHA.Patients who received dosage of 0.5–1.5 mg/Kg/day prednisone for more than 4 weeks didn’t achieved clinical remission were considered to be glucocorticoid-resistant patients.Plasma level of IL-6 was even higher in glucocorticoid-resistant wAIHA patients than in those who responded to glucocorticoid therapy effectively. ROC curve was generated to predict the glucocorticoid efficacy in wAIHA patients. Although the curve is not so satisfactory,plasma level of IL-6 can hopefully become an ideal biological predictor for glucocorticoid efficacy in wAIHA patients. Second-line or third-line treatments, including rituximab or cyclophosphamide should be used in combination with glucocorticoid to control hemolysis in glucocorticoid-resistant wAIHA patients. The onset time of glucocorticoid treatment was positively correlated, and the duration of glucocorticoid treatment was negatively correlated with plasma level of IL-6 in wAIHA. Plasma level of IL-6 not only reflects the degree of hemolysis and disease severity, but also correlates with clinical efficacy and predicts the effect of glucocorticoid therapy.

In vitro cell culture experiments were conducted to explore the effect of IL-6 on the activation and secretion function of B cells in wAIHA patients. Our previous study found that the number of CD5^+^ B cells increased in the peripheral blood of hemolytic episode wAIHA^7^. In this study, IL-6 had no effect on the proportion of CD5^+^B cells in patients with wAIHA. There are currently two hypotheses regarding the origin of CD5^+^ B cells. One is that pro-B cells differentiate in two directions before experiencing immunoglobulin gene rearrangement in the bone marrow and generate two different cell lines (CD5^+^ B cells and CD5^-^ B cells) in the body. Another hypothesis is that CD5^+^B and CD5^-^B cells are derived from the same precursor cell, and CD5^+^B cells are formed by immunoglobulin gene rearrangement after repeated stimulation by specific antigens^13^. IL-6 did not affect expression levels of CD5 on B cells, may indicating that CD5^+^ B cells and CD5^-^ B cells originate from different cell lines in wAIHA patients.

Our previous study has already demonstrated that proportion of CD5^+^CD80^+^/CD19^+^B cells and CD5^+^CD86^+^/CD19^+^ B cells were significantly elevated and correlated with the hemolytic indicators and immune indicators in hemolytic episode wAIHA patients^7^. Activated CD5^+^ B cells in wAIHA patients mainly secrete IL-10 which negatively control the immune response^8^. In this study, we found that both percentage of CD5^+^CD80^+^/CD19^+^ B cells and CD5^-^CD80^+^/CD19^+^ B cells were significantly elevated after IL-6 stimulation. We speculated that IL-6 promoted the activation of B cells in wAIHA. Plasma levels of IL-10 in patients with hemolytic wAIHA were significantly elevated compared with that in HCs. After IL-6 stimulation, intracellular levels of IL-10 in B cells were significantly decreased compared to that in the blank control group. One hypothesis we made was that there were two different B cell subsets exist. For one subset of B cells, it can be abnormally activated and secrete pathogenic auto-antibodies against RBC when stimulated by IL-6 in wAIHA patients. For another subset of B cells,when stimulated by IL-6, it can also be abnormally activated, and secrete IL-10 to negatively regulate immune response in wAIHA patients. A few studies have indicated that regulatory B cells (Bregs) can control the immune response by IL-10 secretion, which is why these B cells subsets are also been named as B10 cells^14^. The phenotype of B10 cells has not been well studied.Activation of IL-6 signaling pathway may not only promote the production of pathogenic auto-antibodies but also promote the production and secretion of IL-10 in Bregs to try to negatively control the immune response in wAIHA patients. However, the specific mechanism by which the IL-6 signaling pathway regulates the production and secretion of IL-10 needs to be further studied and verified.

Compared to CD5^-^B cells, the activation effect of IL-6 on CD5^+^ B cells appeared to be more prominent. Percentage of CD5^+^CD80^+^/CD19^+^B cells and CD5^+^CD86^+^/CD19^+^B cells were higher than CD5^-^CD80^+^/CD19^+^B cells and CD5^-^CD86^+^/CD19^+^B cells in IL-6 stimulation group respectively. However, differences in the expression levels of IL-10 were not statistically significant between CD5^+^ and CD5^-^ B cells after IL-6 stimulation.

A previous study found that CD5^+^ B cells seem to be a B cell subset with an auto-reactivity tendency by secreting a large amount of low-affinity IgM antibodies, which is the body's main source of natural antibodies. When CD5^+^ B cells receive abnormal activation signal stimulation, class switching and affinity maturation occur rapidly, and the secretion of pathogenic antibodies with high affinity mediates the occurrence of AID^15^. However, some studies have found that CD5 can negatively regulate the BCR signaling pathway and promote the secretion of IL-10, negatively regulating the immune response^16^. The activation of the BCR signaling pathway in CD5^-^ B cells can activate three major mitogens-activated protein kinases (MAPKs), namely ERK, JNK, and P38 kinases, as well as Akt kinases and the transcription factors NFAT2 and NF-κB. Compared with CD5^-^ B cells, activation of the BCR signaling pathway in CD5^+^ B cells leads to the sustained activation of ERK kinase and transcription factor NFAT, while the activation of JNK, P38 kinase, and transcription factor NF-κB were inhibited^16,17^. The activation signaling pathways differed between CD5^+^ B cells and CD5^-^ B cells. In this study, we also found that the regulatory mechanisms of IL-6 on CD5^+^ and CD5^-^B cell activity were different, and the specific mechanism still requires further study.

As a pleiotropic cytokine, IL-6 is mainly produced and secreted by monocytes and macrophages in the early stages of infectious disease and plays a critical role in the host defense response by stimulating the activation and differentiation of various immune cells in our body^18^. Increased IL-6 levels have been shown to promote the development of multiple AID and chronic inflammatory responses^19^. We found that plasma level of IL-6 increased in hemolytic wAIHA patients, and was negatively correlated with the efficacy of glucocorticoid, which was consistent with the findings of Lin Xu^20^. IL-6 can be used as an indicator to reflect the severity of wAIHA, predict glucocorticoid efficacy, and guide clinical practice.

As an IL-6 receptor antagonist, tocilizumab is a humanized monoclonal antibody against IL-6R that blocks the signaling pathway by inhibiting the binding of IL-6 to its transmembrane or soluble receptor^21^. In many clinical trials, tocilizumab has been shown to be effective in the treatment of moderate to severely active RA, systemic juvenile idiopathic arthritis, and Castleman's disease, and has been widely utilized in patients with these diseases^22,23^. Clinical studies on tocilizumab in the treatment of systemic lupus erythematosus(SLE), systemic sclerosis, and polymyositis are also in progress^24,25^. Additionally, existing case reports have shown that tocilizumab has significant efficacy in AIHA secondary to SLE or Castleman’s disease, which can improve the hemolytic symptoms of patients and reduce the dosage of glucocorticoid^25,26^.Proportion of CD80^+^/CD5^+^ CD19^+^ B cells and CD80^+^/CD5^-^CD19^+^ B cells in IL-6 + toc stimulation group was lower than that in IL-6 stimulation group. Although there was no statistically significant difference, an obvious trend was observed. We hypothesize that tocilizumab will be another treatment option for hemolytic wAIHA patients by inhibiting B cells activation and secretion pathogenic antibodies.

## Conclusions

Plasma levels of IL-6 were elevated and significantly correlated with disease severity and glucocorticoid efficacy in wAIHA patients. Tocilizumab is expected to be an treatment option for glucocorticoid-resistant wAIHA patients by inhibiting the activation of B cells.

### Supplementary Information

Supplementary Information.

## Data Availability

All the data used to support the findings of this study are available from the corresponding authors upon reasonable request.
