# Supplementary material for: Interleukin 6 exacerbates the progression of warm autoimmune hemolytic anemia by influencing the activity and function of B cells
Source: Sci Rep. 2023 Aug 14;13:13231. doi: 10.1038/s41598-023-40239-w (PMC10425344; doi:10.1038/s41598-023-40239-w)
Supplement: Supplementary file 1 — Supplementary Information. [file 41598_2023_40239_MOESM1_ESM.docx]

**Isotype Controls for Figure 5：**

| 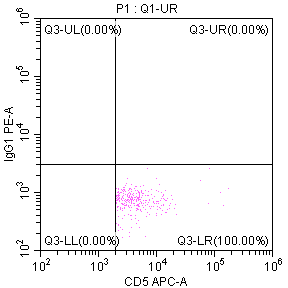 | 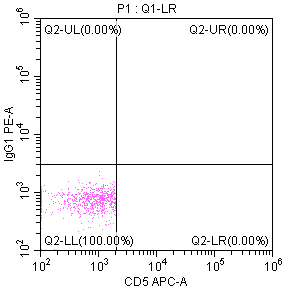 |
| --- | --- |

**Isotype Controls for Figure 6:**

| 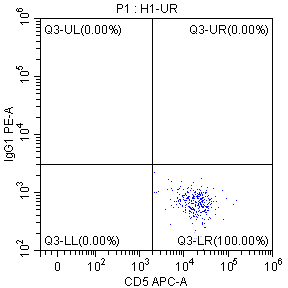 | 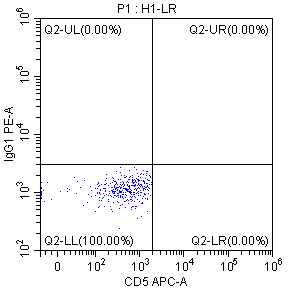 |
| --- | --- |

B cells can not be divided clearly into two cell subsets when labeled with the fluorescent antibody APC-CD5,PE-CD80,PE-CD86 and PE-IL-10. We used isotype control antibodies IgG1-PE to help us distinguish positive cells from negative cells. Finally, we made statistical analysis about the percentage of CD5^+^CD80^+^, CD5^+^CD86^+^,CD5^-^ CD80^+^,CD5^-^CD86^+^,CD5^+^IL-10^+^,CD5^-^IL-10^+^B cells and got our conclusion by comparing the percentage of these cells.
